# Evaluating anthracycline + taxane versus taxane-based chemotherapy in older women with node-negative triple-negative breast cancer: a SEER-Medicare study

**DOI:** 10.1007/s10549-021-06424-z

**Published:** 2021-10-27

**Authors:** Anna R. Schreiber, Jodi Kagihara, Megan Eguchi, Peter Kabos, Christine M. Fisher, Elisabeth Meyer, Elizabeth Molina, Lavanya Kondapalli, Cathy J. Bradley, Jennifer R. Diamond

**Affiliations:** 1 Department of Medicine, University of Colorado Anschutz Medical Campus, Aurora, USA; 2 Division of Medical Oncology, University of Colorado Anschutz Medical Campus, 12801 East 17th Ave, Mailstop 8117, Aurora, CO 80045 USA; 3 School of Public Health, Department of Health Systems, Management, and Policy, University of Colorado Anschutz Medical Campus, Aurora, USA; 4 Department of Radiation Oncology, University of Colorado Anschutz Medical Campus, Aurora, USA; 5 Division of Cardiology, University of Colorado Anschutz Medical Campus, Aurora, USA

**Keywords:** Triple-negative breast cancer (TNBC), Adjuvant chemotherapy, Surveillance, Epidemiology, and End Results (SEER), Node-negative, Anthracycline

## Abstract

**Purpose:**

Adjuvant chemotherapy reduces recurrence in early-stage triple-negative breast cancer (TNBC). However, data are lacking evaluating anthracycline + taxane (ATAX) versus taxane-based (TAX) chemotherapy in older women with node-negative TNBC, as they are often excluded from trials. The purpose of this study was to evaluate the effect of adjuvant ATAX versus TAX on cancer-specific (CSS) and overall survival (OS) in older patients with node-negative TNBC.

**Patients and methods:**

Using the SEER-Medicare database, we selected patients aged ≥ 66 years diagnosed with Stage T1-4N0M0 TNBC between 2010 and 2015 (*N* = 3348). Kaplan–Meier survival curves and adjusted Cox proportional hazards models were used to estimate 3-year OS and CSS. Multivariant Cox regression analysis was used to identify independent factors associated with use of ATAX compared to TAX.

**Results:**

Approximately half (*N* = 1679) of patients identified received chemotherapy and of these, 58.6% (*N* = 984) received TAX, 25.0% (*N* = 420) received ATAX, and 16.4% (*N* = 275) received another regimen. Three-year CSS and OS was improved with any adjuvant chemotherapy from 88.9 to 92.2% (*p* = 0.0018) for CSS and 77.2% to 88.6% for OS (*p* < 0.0001). In contrast, treatment with ATAX compared to TAX was associated with inferior 3-year CSS and OS. Three-year CSS was 93.7% with TAX compared to 89.8% (*p* = 0.048) for ATAX and OS was 91.0% for TAX and 86.4% for ATAX (*p* = 0.032).

**Conclusion:**

While adjuvant chemotherapy was associated with improved clinical outcomes, the administration of ATAX compared to TAX was associated with inferior 3-year OS and CSS in older women with node-negative TNBC. The use of adjuvant ATAX should be considered carefully in this patient population.

**Supplementary Information:**

The online version contains supplementary material available at 10.1007/s10549-021-06424-z.

## Background

Breast cancer is the most common cancer diagnosed in women with approximately 280,000 new cases in the USA in 2020 [1]. Triple-negative breast cancer (TNBC) accounts for 10–15% of cases and is defined by a lack of expression of the estrogen receptor (ER), progesterone receptor (PR), and lack of human epidermal growth factor 2 (HER2) over-expression [1]. As such, endocrine and HER2-targeted therapies are ineffective and cytotoxic chemotherapy remains the mainstay of neoadjuvant or adjuvant treatment to prevent recurrence even in patients diagnosed with early-stage, node-negative TNBC [2, 3].

Patients diagnosed with TNBC have a higher risk of metastatic recurrence and inferior disease-free and overall survival (OS) compared to stage-matched patients with other breast cancer subtypes [3]. Adjuvant chemotherapy proportionally provides more benefit in patients with TNBC compared to ER + , HER2− breast cancer in preventing metastatic recurrence and adjuvant chemotherapy is generally recommended for patients with early-stage TNBC and tumors > 0.5 cm or node-positive disease [4, 5].

While TNBC is more common in younger women, approximately one third of patients with TNBC are > 65 years old [6]. Older patients remain under-represented in adjuvant chemotherapy clinical trials [7]. Given the lack of prospective clinical trial data, treating this elderly population remains a clinical challenge and there are risks to both over- and under-treatment. On the one hand, functional status does not always correlate with chemotherapy tolerance and on the other, older patients with breast cancer and a preserved functional status are often treated with less aggressive chemotherapy regimens despite some studies suggesting that these patients do benefit from standard chemotherapy [8–11].

Chemotherapy regimens containing an anthracycline and a taxane reduce the risk of breast cancer recurrence, breast cancer mortality, and overall mortality compared to other cytotoxic regimens as demonstrated in the Early Breast Cancer Trialists’ Collaborative Group (EBCTCG) meta-analysis [5]. The ABC trials confirmed this finding prospectively in patients with high-risk node-negative or node-positive HER2− breast cancer, where there was a statistically significant improvement in four-year invasive disease-free survival (iDFS) with doxorubicin and cyclophosphamide with a taxane compared to docetaxel and cyclophosphamide (90.7% vs 88.2%, *p* = 0.04), although the absolute benefit was small in patients with node-negative TNBC (four-year iDFS of 89.5% vs 87.0% for an absolute change of 2.5%) [12]. Additionally, the majority of patients enrolled in the ABC trials were younger than age 66 years and presumably at lower risk of cardiac toxicity associated with anthracyclines that increases with age [12, 13].

The benefit of the addition of an anthracycline to a taxane-based chemotherapy regimen in older patients with TNBC remains understudied with current available clinical trial data [14]. The purpose of this study was to evaluate the benefit of adjuvant chemotherapy, and specifically focus on the administration of an anthracycline + taxane (ATAX) versus taxane-based (TAX) regimen, on cancer-specific survival (CSS) and OS in older women with node-negative TNBC using data from linked Surveillance, Epidemiology, and End Results (SEER)-registry Medicare claim files. In addition, we sought to explore whether age, tumor size, and the presence of comorbidities impacted selection of ATAX versus TAX in clinical practice.

## Methods

### Data

The SEER-Medicare database links SEER registry data with Medicare enrollment and claims data. The SEER registries provide comprehensive population-based data on cancer incidence and survival in the USA and include approximately 35% of the US population [15]. The SEER registries include data on demographics, date of diagnosis, cancer site, and stage at diagnosis. The Medicare data include vital status and health service utilization information for Medicare fee-for-service beneficiaries [16, 17]. The linkage of these two data sources allowed analysis of breast cancer subtype, stage at diagnosis, demographics, cardiac comorbidities, receipt of chemotherapy drugs, cancer recurrence, and survival for individual patients. This study was conducted following local institutional review board approval.

### Sample

**Fig. 1:**
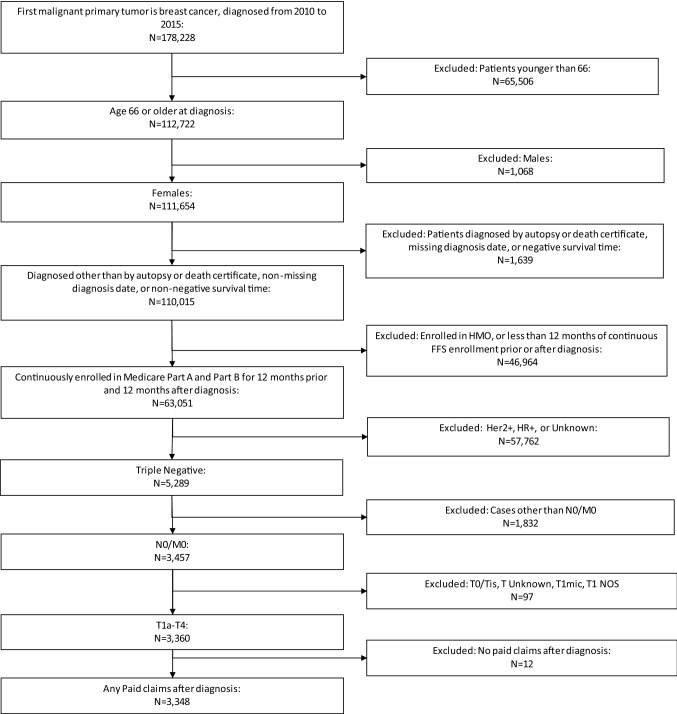
Sample selection. Newly diagnosed node-negative TNBC in women age 66 or older between 2010 and 2015 in SEER-Medicare

We selected female beneficiaries aged 66 years or older diagnosed with primary American Joint Committee on Cancer (AJCC) 7th version Stage T1-4N0M0 invasive TNBC between 2010 and 2015 (N = 3348). We included patients continuously enrolled in Medicare Part A and B for at least 12 months prior to and after their initial diagnosis. Patients were excluded if the month of diagnosis was unknown, diagnosis was by autopsy or death certificate, or patients had T1mic or T1 NOS disease. In addition, we excluded 12 patients who had no paid claims after diagnosis. The sample selection process is shown in Fig. 1.

Data extracted included age, ethnicity, geographic region, tumor stage, Charlson Comorbidity Index (CCI), type of facility visited, cancer treatment, and history of cardiac and non-cardiac conditions. CCI is a stratification tool that predicts 10-year survival in patients with comorbid conditions such as dementia, liver disease, and heart disease.

Prior cardiac conditions included acute myocardial infarction, atrial fibrillation, diabetes, heart failure, hyperlipidemia, hypertension, ischemic heart disease, peripheral vascular disease, tobacco use, and history of myocardial infarction (Supplemental Table S1) [18]. Patients with T1a and T1b disease and T3 and T4 disease were analyzed together in T1a/b and T3/T4 groups due to limited patient numbers in these groups.

### Chemotherapy administration

The administration of neoadjuvant or adjuvant chemotherapy was determined using CPT and HCPCS procedure codes for commonly used chemotherapy drugs. Patients were identified as receiving ATAX if they received doxorubicin or epirubicin plus paclitaxel, docetaxel, or nab-paclitaxel and as receiving TAX if they received docetaxel, paclitaxel, or nab-paclitaxel without doxorubicin or epirubicin. Receipt of other drugs in combination with these agents, including cyclophosphamide, was not specifically captured.

### Statistical analysis

Descriptive statistics were used to summarize demographic features across the treatment groups. Chi-square analysis was used to determine statistical significance of differences in descriptive characteristics across treatment groups. Overall survival was defined as death due to any cause determined by the time from the month of diagnosis to death. Cancer-specific survival was defined as death from cancer determined by the time from the month of diagnosis to death. Kaplan–Meier 3-year all-cause and cancer-specific survival curves were generated for treatment groups and for patients by age groups. OS and CSS between chemotherapy regimens was estimated using adjusted Cox proportional hazards models. Forest plots were generated using multivariate analysis and adjusted Cox proportional hazards models. Logistic regression was used to estimate odds ratios (OR) and 95% confidence intervals (CIs) for the association between covariates and treatment groups.

## Results

### Cohort characteristics

Table 1 reports characteristics of the cohort. Patients were aged 66–75 (56.8%) and 76 years or older (43.2%). The majority of patients were white (77.6%) and 13% were black. Most patients were diagnosed in the West, followed by the South, Northeast, and Midwest regions of the country. The majority of patients had T1c (35.6%) or T2 (34.1%) tumors and approximately half (46.6%) had a score of one or greater on the CCI. Patients more commonly visited teaching hospitals followed by National Cancer Institute (NCI) centers. Surgery, radiation therapy (RT), and chemotherapy were the most common treatment. The majority of patients (83.1%) had prior cardiac conditions; however, only 27.8% had a prior non-cardiac condition.

**Table 1:** Descriptive characteristics and statistics, women diagnosed with TNBC by chemotherapy administration, SEER-Medicare 2010–2015

All patients					Patients who received ATAX and TAX			
Variable	Overall	No chemotherapy	Any chemotherapy	*p*-value	Overall	Taxane	Taxane + anthracycline	*p*-value
All patients	3348	1669	1679		1404	984	420	
Age category								
66 to 75	1903 (56.8)	603 (36.1)	1300 (77.4)	< 0.0001	1134 (80.8)	747 (75.9)	387 (92.1)	< 0.0001
76 and older	1445 (43.2)	1066 (63.9)	379 (22.6)		270 (19.2)	237 (24.1)	33 (7.9)	
Ethnicity								
White	2597 (77.6)	1296 (77.7)	1301 (77.5)	0.8303	1087 (77.4)	761 (77.3)	326 (77.6)	0.7318
Black	435 (13.0)	212 (12.7)	223 (13.3)		185 (13.2)	127 (12.9)	58 (13.8)	
Other	316 (9.4)	161 (9.7)	155 (9.2)		132 (9.4)	96 (9.8)	36 (8.6)	
Region at diagnosis								
Northeast	666 (19.9)	323 (19.4)	343 (20.4)	0.4511	272 (19.4)	179 (18.2)	93 (22.1)	0.0152
Midwest	444 (13.3)	235 (14.1)	209 (12.5)		177 (12.6)	114 (11.6)	63 (15.0)	
South	839 (25.1)	424 (25.4)	415 (24.7)		341 (24.3)	235 (23.9)	106 (25.2)	
West	1399 (41.8)	687 (41.2)	712 (42.4)		614 (43.7)	456 (46.3)	158 (37.6)	
Tumor size								
T1a/T1b	792 (23.7)	535 (32.1)	257 (15.3)	< 0.0001	202 (14.4)	167 (17.0)	35 (8.3)	< 0.0001
T1c	1193 (35.6)	531 (31.8)	662 (39.4)		566 (40.3)	425(43.2)	141 (33.6)	
T2	1143 (34.1)	492 (29.5)	651 (38.8)		553 (39.4)	347 (35.3)	206 (49.1)	
T3/T4	220 (6.6)	111 (6.7)	109 (6.5)		83 (5.9)	45 (4.6)	38 (9.1)	
Charlson Comorbidity Index								
0	1788 (53.4)	793 (47.5)	995 (59.3)	< 0.0001	859 (61.2)	573 (58.2)	286 (68.1)	0.0012
1	793 (23.7)	398 (23.8)	395 (23.5)		324 (23.1)	238 (24.2)	86 (20.5)	
2 or more	767 (22.9)	478 (28.6)	289 (17.2)		221 (15.7)	173 (17.6)	48 (11.4)	
Facilities visited in 6 months								
NCI Center	484 (14.5)	216 (12.9)	268 (16.0)	0.0041	222 (15.8)	143 (14.5)	79 (18.8)	0.1305
Teaching Hospital	1820 (54.4)	895 (53.6)	925 (55.1)		786 (56.0)	558 (56.7)	228 (54.3)	
Other	1044 (31.2)	558 (33.4)	486 (29.0)		396 (28.2)	283 (28.8)	113 (26.9)	
Treatment								
Surgery alone	823 (24.6)	823 (49.3)	0	< 0.0001				
Surgery and RT	846 (25.3)	846 (50.7)	0					
Surgery and chemotherapy	516 (15.4)	0	516 (30.7)		404 (28.8)	286 (29.1)	118 (28.1)	0.7132
Surgery, RT, and chemotherapy	1163 (34.7)	0	1163 (69.3)		1000 (71.2)	698 (70.9)	302 (71.9)	
Prior heart failure								
No	3085 (92.1)	1486 (89.0)	1599 (95.2)	< 0.0001	1342 (95.6)	935 (95.0)	407 (96.9)	0.1156
Yes	266 (7.9)	183 (11.0)	80 (4.8)		62 (4.4)	49 (5.0)	13 (3.1)	
Prior ischemic heart disease								
No	2713 (81.0)	1294 (77.5)	1419 (84.5)	< 0.0001	1199 (85.4)	826 (83.9)	373 (88.8)	0.0181
Yes	635 (19.0)	375 (22.5)	260 (15.5)		205 (14.6)	158 (16.1)	47 (11.2)	
Any prior cardiac conditions								
No	533 (15.9)	217 (13.0)	316 (18.8)	< 0.0001	266 (19.0)	169 (17.2)	92 (23.1)	0.0095
Yes	2815 (83.1)	1452 (87.0)	1363 (81.2)		1138 (81.1)	815 (82.8)	323 (76.9)	
Any prior non-cardiac conditions								
No	2417 (72.2)	1110 (66.5)	1307 (77.8)	< 0.0001	1117 (79.6)	765 (77.7)	352 (83.8)	0.0099
Yes	931 (27.8)	559 (33.5)	372 (22.2)		287 (20.4)	219 (22.3)	68 (16.2)	

Statistics are unweighted column percentages

NCI Center (National Cancer Institute (NCI)-Designated Cancer Center), radiation therapy (RT), tumor size is AJCC 7th edition

### Chemotherapy administration

Of the 3348 patients included in the cohort, 1679 (50.1%) received chemotherapy with any agent. Patients aged 66 to 75 years received chemotherapy at a higher frequency (77.4%) compared to patients aged 76 and older (22.6%). The proportion of patients aged 66 or older receiving chemotherapy was highest in patients with T2 disease, with 32.4% of patients with T1a/T1b disease, 55.5% with T1c disease, 57% with T2 disease, and 50% with T3/T4 disease receiving chemotherapy. As expected, chemotherapy was less frequently administered to patients with a higher CCI, prior heart failure, ischemic heart disease, and any prior cardiac or non-cardiac condition. Of the patients who received chemotherapy, a higher proportion also received RT in addition to chemotherapy and surgery (69.3% vs 30.7%). Ethnicity, region of diagnosis, and facilities visited were similar between the two groups.

Of the 1,679 patients who received chemotherapy, 984 (58.6%) received TAX, 420 (25.0%) received ATAX, and 275 (16.4%) received another chemotherapy regimen. The most common other chemotherapy regimen received was cyclophosphamide, methotrexate, fluorouracil (CMF) (32.4%). Of the patients who received either ATAX or TAX, we found a higher proportion of older patients received TAX (65.9% of patients aged 66–75 and 87.8% of patients 76 and older). The majority of patients receiving ATAX had T2 tumors (49%) and the majority of patients receiving TAX had T1c tumors (43%). A higher proportion of patients with CCI ≥ 2, prior heart failure, prior ischemic heart disease, and any prior cardiac or non-cardiac condition received TAX. Ethnicity, region of the country, type of facility where care was received, and treatment were similar between groups.

### Independent factors associated with the use of adjuvant chemotherapy

Table 2 reports estimates from a logistic regression model used to predict variables associated with chemotherapy use. Patients aged 76 and older were less likely to receive any chemotherapy regimen with an OR of 0.16 (95% CI 0.13 to 0.18, *p* < 0.0001) relative to women aged 66 to 75. Tumor stage was also positively and statistically significantly associated with chemotherapy use and women with T2 and T3/T4 tumors were approximately 4 times more likely to receive chemotherapy compared to women with T1a/T1b tumors. The presence of prior cardiac conditions was not statistically associated with receipt of chemotherapy; however, prior non-cardiac conditions were negatively and statistically significantly associated with chemotherapy receipt (OR 0.63, 95% CI 0.53 to 0.75, *p* < 0.0001). Ethnicity, region, and facility type were not statistically significantly different between the chemotherapy and no chemotherapy group.

**Table 2:** Logistic regression analysis estimating OR of chemotherapy vs no chemotherapy and taxane + anthracycline-containing regimen vs taxane-containing regimen across variables, SEER-Medicare 2010–2015

Variable	Chemotherapy vs no chemotherapy *N* = 3348		Taxane + anthracycline vs taxane *N* = 1404	
	OR (95% CI)	*p*-value	OR (95% CI)	*p*-value
Age at diagnosis				
66 to 75 (ref)				
76 and older vs 66 to 75	0.16 (0.13, 0.18)	< 0.0001	0.25 (0.17, 0.38)	< 0.0001
Ethnicity				
White (ref)				
Black vs White	1.01 (0.79, 1.31)	0.9128	1.04 (0.71, 1.52)	0.8514
Other vs White	0.88 (0.67, 1.16)	0.3643	1.02 (0.66, 1.58)	0.9271
Region				
West (ref)				
Midwest vs West	0.91 (0.70, 1.19)	0.4917	1.84 (1.23, 2.75)	0.0031
Northeast vs West	1.12 (0.90, 1.41)	0.3183	1.75 (1.24, 2.48)	0.0014
South vs West	0.91 (0.74, 1.13)	0.3998	1.32 (0.94, 1.84)	0.1077
Tumor size				
T1a/T1b (ref)				
T1c vs T1a/T1b	3.40 (2.75, 4.20)	< 0.0001	1.67 (1.10, 2.55)	0.0171
T2 vs T1a/T1b	4.30 (3.45, 5.35)	< 0.0001	3.33 (2.19, 5.06)	< 0.0001
T3/T4 vs T1a/T1b	4.16 (2.93, 5.92)	< 0.0001	5.19 (2.85, 9.46)	< 0.0001
Facility type				
Other (ref)				
NCI center vs other	1.11 (0.86, 1.43)	0.4329	1.14 (0.78, 1.68)	0.5075
Teaching hospital vs other	0.99 (0.82, 1.19)	0.9285	0.75 (0.56, 1.02)	0.0626
Prior cardiac conditions				
No (ref)				
Yes vs no	0.98 (0.78, 1.21)	0.8196	0.76 (0.56, 1.04)	0.0822
Prior non-cardiac conditions				
No (ref)				
Yes vs no	0.63 (0.53, 0.75)	< 0.0001	0.73 (0.53, 1.00)	0.0528

Statistics are unweighted column percentages

NCI Center (National Cancer Institute (NCI)-Designated Cancer Center), radiation therapy (RT), odds ratio (OR), reference (ref), tumor size is AJCC 7th edition

When comparing ATAX to TAX, women aged 76 and older were less likely to receive ATAX with an OR of 0.25 (95% CI 0.17 to 0.38, *p* < 0.0001) relative to women aged 66 to 75. Higher tumor stage was positively and statistically significantly associated with use of ATAX compared to TAX and women with T3/T4 tumors were over 5 times as likely to receive ATAX compared to women with T1a/T1b tumors. Prior cardiac and non-cardiac conditions were negatively associated with ATAX administration, although this trend did not reach statistical significance. Patients diagnosed in the Northeast and Midwest were more likely to be treated with ATAX compared to those in the West. There were no significant differences in ethnicity or facility type between treatment groups.

### ATAX treatment is associated with inferior survival in older women

**Fig. 2:**
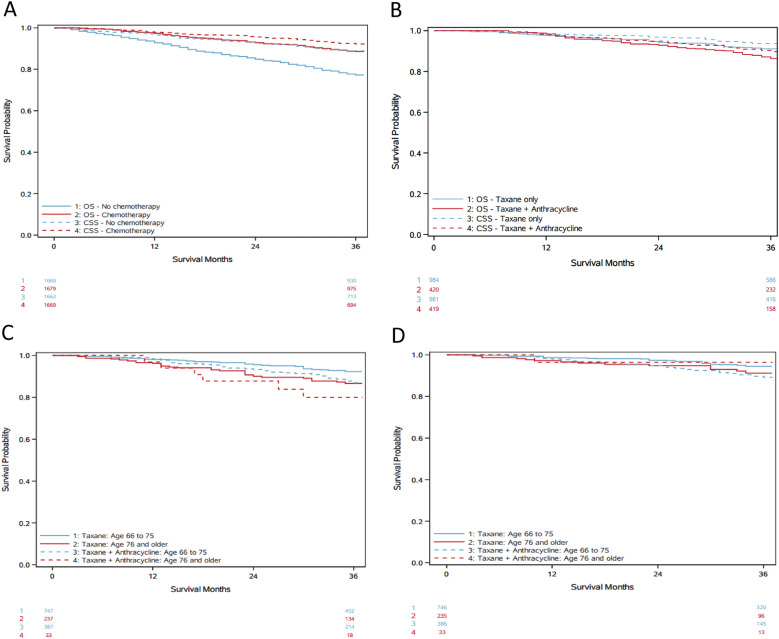
Kaplan–Meier survival curves demonstrating survival probability over 3 years. Total number at risk is reflected at 0 months and 36 months for all curves. (**A**) Cancer-specific survival (CSS) and overall survival (OS) for patients who received chemotherapy and those who did not. (**B**) CSS and OS for patients who received anthracycline + taxane-containing (ATAX) chemotherapy vs taxane-containing (TAX). (**C**) OS distributed by those who received ATAX vs TAX aged 66 to 75 and 76 and older. (**D**) CSS distributed by those who received ATAX vs TAX aged 66 to 75 and 76 and older

We performed a univariate Kaplan–Meier analysis of survival estimating CSS and OS over 3 years (Fig. 2). There was a statistically significant improvement in CSS and OS in patients who received any form of adjuvant chemotherapy (Fig. 2A). Three-year CSS was 92.2% for patients who received adjuvant chemotherapy compared to 88.9% (*p* = 0.0018) for those who did not. Similarly, 3-year OS for patients who received adjuvant chemotherapy was 88.6% compared to 77.2% (*p* < 0.0001) for those who did not.

For patients who received adjuvant chemotherapy with TAX or ATAX, there was a statistically significant improvement in 3-year CSS and OS for patients who received TAX compared to ATAX (Fig. 2B). Three-year CSS was 93.7% for patients who received TAX compared to 89.8% for patients who received ATAX (*p* = 0.048). Three-year OS for patients who received TAX was 91.0% compared to 86.4% for patients who received ATAX (*p* = 0.032). There was also a statistically significant improvement in 3-year CSS and OS for patients aged 66 to 75 years who received TAX compared to ATAX (Fig. 2C, D). Three-year CSS was 94.4% in patients aged 66–75 who received TAX compared to 89.2% (*p* = 0.0105) for those who received ATAX and OS was 92.4% for TAX and 87.0% for ATAX (*p* = 0.0113). For patients aged 76 and older, there was no statistically significant difference in 3-year CSS and OS.

### Analysis of overall survival controlling for covariates

**Fig. 3:**
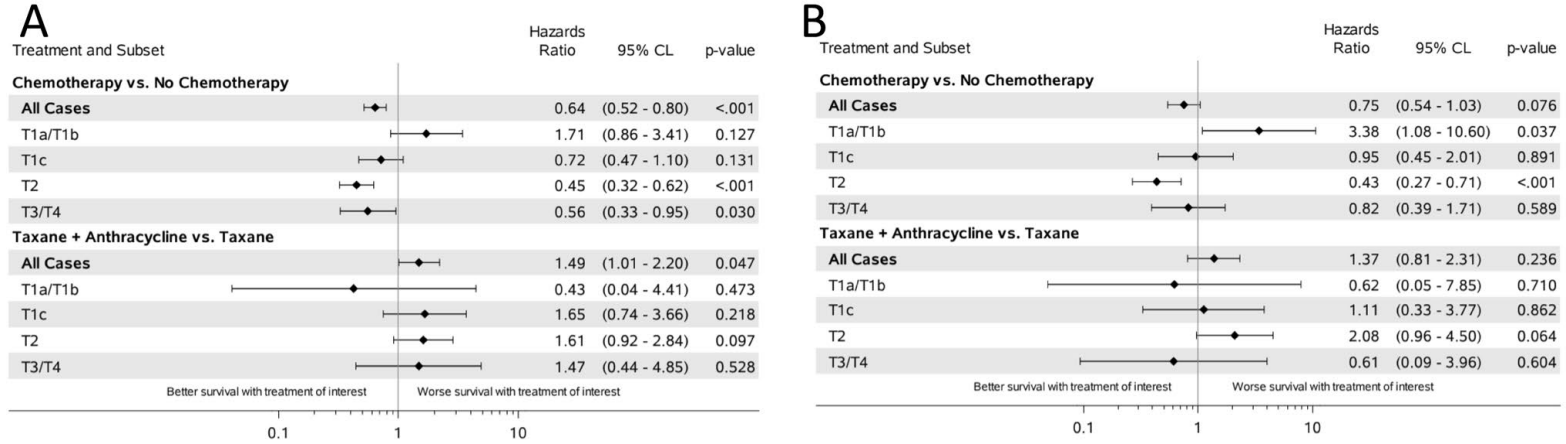
Forest plots for multivariate analysis of (**A**) overall survival (OS) and (**B**) cancer-specific survival (CSS). Hazard ratios shown overall and by stage after reflecting for all other covariates

Cox proportional hazard estimations, controlling for all covariates, are depicted in Fig. 3. Treatment with any chemotherapy regimen was associated with improved OS with a hazard ratio (HR) of 0.64 (95% CI 0.52–0.80, *p* < 0.001) with greater benefit in patients with higher T stage. When compared to TAX, ATAX did not improve OS across all cases (HR 1.49, 95% CI 1.01–2.20, *p* = 0.047). When controlling for T stage, treatment with ATAX versus TAX was not associated with a statistically significant improvement in OS. In fact, we observed a trend toward inferior OS with ATAX compared to TAX at higher T stages (T1c: HR 1.65, 95% CI 0.74–3.66, *p* = 0.218; T2: HR 1.61, 95% CI 0.92–2.84, *p* = 0.097; T3/4: HR 1.47, 95% CI 0.44–4.85, *p* = 0.528).

Trends were similar for CSS with benefit observed for any chemotherapy regimen administered (HR 0.75, 95% CI 0.54–1.03, *p* = 0.076) (Fig. 3). The administration of ATAX compared to TAX did not result in a statistically significant improvement in CSS in any T stage subgroup. There was no subset that clearly seemed to benefit from ATAX compared to TAX with regard to OS or CSS.

## Discussion

We retrospectively evaluated practice patterns and clinical outcomes for older women diagnosed with node-negative TNBC using the SEER-Medicare database. We conducted this study, as the question is not asked or answered in prospective trials, as older patients are under-represented, or plainly excluded due to comorbid conditions. Due to lack of randomized control trials in this patient population, retrospective analysis is the best data available to guide decisions in this elderly population. We specifically set out to evaluate the benefit of adjuvant chemotherapy in these patients and more specifically the benefit of ATAX compared to TAX.

Consistent with prior reports, our study confirmed the benefit of adjuvant chemotherapy in older patients with node-negative TNBC based on an observed improvement in CSS (92.2% versus 88.9%, *p* = 0.0018) and OS (88.6% versus 77.2%, *p* < 0.0001) [9, 19]. Our study is unique in evaluating clinical outcomes for these patients receiving ATAX compared to TAX. We found that patients who received ATAX had inferior CSS (89.8% versus 93.7%, *p* = 0.048) and OS (86.4% versus 91.0%, *p* = 0.032) compared to patients receiving TAX. Furthermore, when controlling for covariates including T stage, OS across all cases was worse with treatment of ATAX compared to TAX (HR 1.49, 95% CI 1.01–2.20, *p* = 0.047). As expected, patients who received ATAX were younger and had fewer comorbidities, but still had inferior OS compared to those who received TAX. Our results do not account for a longer follow-up period and likely a further increase in cardiac complications.

We noted a significantly inferior 3-year CSS (89.2% versus 94.4%, *p* = 0.0105) and OS (87.0% versus 92.4%, *p* = 0.0113) in early-stage node-negative TNBC patients aged 66–75 when treated with ATAX compared to TAX. These results were not consistent in patients > 76 years old; however, sample size was limited in this group and the same trend was observed. We performed Cox proportional hazard estimations controlling for T stage and the administration of ATAX compared to TAX was not associated with a statistically significant improvement in CSS or OS in any T stage group. This suggests that the inferior CSS and OS observed in patients receiving ATAX compared to TAX was not due more patients with higher risk disease treated with ATAX. The majority of patients who received ATAX or TAX in our study had T1c or T2 disease (in total 79.7%, N = 1119) and smaller numbers of patients with T1a/b or T3/4 disease were included, limiting our conclusions in these populations.

In the ABC trials, the absolute benefit of ATAX compared to TAX was small for patients with node-negative TNBC (4-year iDFS change of 2.5%) [12]. While the addition of anthracyclines to taxane-based chemotherapy regimens does improve clinical outcomes in patients with early-stage HER2-negative breast cancer, the benefit needs to be balanced with the risk of toxicity [20]. It is likely that treatment-related toxicity played a role in the inferior OS observed in patients treated with ATAX compared to TAX. When compared to TAX, ATAX is generally a longer treatment regimen (16–20 weeks depending on if the paclitaxel is administered dose dense every 2 weeks × 4 or weekly × 12 weeks) compared to docetaxel cyclophosphamide (administered every 3 weeks × 4 for a total of 12 weeks). ATAX is also a 3-drug regimen compared to a 2-drug regimen with TAX and is often administered on a dose-dense schedule. In addition, treatment with an anthracycline can be more toxic. When comparing treatment with taxane-based therapy to an anthracycline and taxane therapy, one review article found that the anthracycline-containing group had an increased risk of toxicity [21]. Patients in the anthracycline group were found to have higher grade 3 or 4 mucositis, thrombocytopenia, and neuropathy when compared to the taxane-based group [21]. It may be difficult for an elderly population to overcome the higher percentage of toxicities associated with ATAX, leading to inferior outcomes.

Patients who had prior cardiac conditions in our study were less likely to be treated with an anthracycline which is consistent with current guidelines [22]. In prior studies, while patients receiving ATAX were in general younger and more likely to be without cardiac comorbidities, patients older than 65 were approximately twice as likely to experience anthracycline-induced congestive heart failure (CHF) compared to younger patients [23, 24]. It is possible that patients who received ATAX had worse outcomes because they developed cardiac conditions, a risk that may be less significant in patients younger than 65 years of age. However, this statement is just a theory as it is difficult to draw firm conclusions without supporting cardiac data. Future studies evaluating cardiac outcomes following treatment with ATAX compared to TAX could be of interest.

Despite multiple studies demonstrating the benefit of adjuvant chemotherapy in patients with early-stage TNBC, limited data exist to guide decision-making in older patients [5, 12]. In the ABC trials, for example, only 29% of patients were older than age 60 [12]. Geriatric assessment tools can be useful in evaluating older patients and making treatment decisions that are not based solely on patient age [25]. Previous studies are mixed in reporting tolerability for anthracyclines and taxanes in elderly patients with some reporting major short-term toxicity and others reporting general tolerability [11, 26, 27]. Furthermore, older patients are more likely to have pre-existing cardiac conditions or cardiac risk factors that can increase risk of cardiotoxicity [28, 29]. All of these factors can make selecting a treatment regimen difficult.

In our study, adjuvant chemotherapy was administered to approximately three-fourths of patients aged 66–75 and < 30% of patients older than 76 years. Not surprisingly, patients were also more likely to receive chemotherapy if they had larger tumors. Patients located in Northeast and the Midwest were more likely to receive ATAX versus TAX when compared to the West. This supports that there are regional differences in chemotherapy preference, which may be driven by large academic centers. Patients with non-cardiac comorbidities were less likely to receive any adjuvant chemotherapy regimen. This may be explained by a perceived higher risk of treatment complications and a shorter life expectancy by the treating provider. Administration of adjuvant chemotherapy was associated with improved CSS and OS in this elderly patient population, highlighting the benefit in selected patients. Similarly, a recent study found benefit in the addition of chemotherapy to patients older than 70 years with node-positive and node-negative TNBC [9]. The survival benefit shown in these results highlights the importance of considering chemotherapy in this elderly, node-negative TNBC population.

This study has several limitations: First, there is likely inherent bias in that patients projected to have a longer life expectancy were more likely to receive adjuvant chemotherapy. Second, although we used the most recent SEER-Medicare data, the data may not reflect the most recent practices and are specific to the fee-for-service population. In addition, we do not have information of cause of death, toxicity, or quality of life. Nevertheless, this study supports consideration of adjuvant chemotherapy in elderly patients without significant co-morbid conditions who are expected to have a sufficient life expectancy to obtain benefit and brings pause to the use of ATAX in this population.

## Conclusion

To our knowledge, this is the largest cohort study to evaluate administration of ATAX versus TAX and examine clinical outcomes in older patient treated for early-stage TNBC. Similar to other studies, this analysis supports the consideration of chemotherapy in an elderly, node-negative TNBC population. What differs from other studies is we did not find a survival benefit with use of ATAX compared to TAX; this differed from the ABC trials and EBCTCG meta-analysis which looked at a wider and younger age range [5, 12]. Providers should approach elderly, node-negative TNBC patients cautiously and consider use of TAX in place of an anthracycline-containing regimen. Further efforts should be put toward developing large, randomized trials that include elderly patients to better understand the benefits and pitfalls of therapies in elderly patients.

## Supplementary Information

Below is the link to the electronic supplementary material.

Supplementary file1 (DOCX 16 KB)

## Data Availability

The datasets analyzed during the current study are available from the National Cancer Institute’s Surveillance, Epidemiology, and End Results program database: https://seer.cancer.gov/data/access.html.

## Abbreviations

TNBC: Triple-negative breast cancer
ER: Estrogen receptor
PR: Progesterone receptor
HER2: Human epidermal growth factor 2
OS: Overall survival
EBCTCG: Early Breast Cancer Trialists’ Collaborative Group
iDFS: Invasive disease-free survival
ATAX: Anthracycline + taxane chemotherapy
TAX: Taxane-based chemotherapy
CSS: Cancer-specific survival
SEER: Surveillance, Epidemiology, and End Results
AJCC: American Joint Committee on Cancer
CCI: Charlson comorbidity index
OR: Odds ratio
CI: Confidence interval
NCI: National Cancer Institute
RT: Radiation therapy
CMF: Cyclophosphamide, methotrexate, fluorouracil
HR: Hazard ratio
CHF: Congestive heart failure

## References

[1] Cancer Stat Facts: Female Breast Cancer Subtypes (2020) https://seer.cancer.gov/statfacts/html/breast-subtypes.html

[2] Engebraaten O, Vollan HKM, Børresen-Dale AL (2013). Triple-negative breast cancer and the need for new therapeutic targets. Am J Pathol.

[3] Kumar P, Aggarwal R (2016). An overview of triple-negative breast cancer. Arch Gynecol Obstet.

[4] Rampurwala MM, Rocque GB, Burkard ME (2014). Update on adjuvant chemotherapy for early breast cancer. Breast Cancer.

[5] Peto R, Davies C, Godwin J, Gray R, Pan HC, Clarke M, Cutter D, Darby S, McGale P, Taylor C (2012). Comparisons between different polychemotherapy regimens for early breast cancer: meta-analyses of long-term outcome among 100,000 women in 123 randomised trials. Lancet.

[6] Howlader N, Altekruse SF, Li CI, Chen VW, Clarke CA, Ries LA, Cronin KA (2014). US incidence of breast cancer subtypes defined by joint hormone receptor and HER2 status. J Natl Cancer Inst.

[7] Hutchins LF, Unger JM, Crowley JJ, Coltman CA, Albain KS (1999). Underrepresentation of patients 65 years of age or older in cancer-treatment trials. N Engl J Med.

[8] von Minckwitz G, Conrad B, Reimer T, Decker T, Eidtmann H, Eiermann W, Hackmann J, Möbus V, Marmé F, Potenberg J (2015). A randomized phase 2 study comparing EC or CMF versus nab-paclitaxel plus capecitabine as adjuvant chemotherapy for nonfrail elderly patients with moderate to high-risk early breast cancer (ICE II-GBG 52). Cancer.

[9] Crozier JA, Pezzi TA, Hodge C, Janeva S, Lesnikoski BA, Samiian L, Devereaux A, Hammond W, Audisio RA, Pezzi CM (2020). Addition of chemotherapy to local therapy in women aged 70 years or older with triple-negative breast cancer: a propensity-matched analysis. Lancet Oncol.

[10] Giordano SH, Hortobagyi GN, Kau SW, Theriault RL, Bondy ML (2005). Breast cancer treatment guidelines in older women. J Clin Oncol.

[11] Muss HB, Berry DA, Cirrincione CT, Theodoulou M, Mauer AM, Kornblith AB, Partridge AH, Dressler LG, Cohen HJ, Becker HP (2009). Adjuvant chemotherapy in older women with early-stage breast cancer. N Engl J Med.

[12] Blum JL, Flynn PJ, Yothers G, Asmar L, Geyer CE, Jacobs SA, Robert NJ, Hopkins JO, O'Shaughnessy JA, Dang CT (2017). Anthracyclines in early breast cancer: the ABC trials-USOR 06–090, NSABP B-46-I/USOR 07132, and NSABP B-49 (NRG oncology). J Clin Oncol.

[13] Neuendorff NR, Loh KP, Mims AS, Christofyllakis K, Soo WK, Bölükbasi B, Oñoro-Algar C, Hundley WG, Klepin HD (2020). Anthracycline-related cardiotoxicity in older patients with acute myeloid leukemia: a Young SIOG review paper. Blood Adv.

[14] Payne DL, Nohria A (2017). Prevention of Chemotherapy Induced Cardiomyopathy. Curr Heart Fail Rep.

[15] SEER Incidence Data (1975–2017) https://seer.cancer.gov/data/

[16] Sciences NCIDoCCaP: SEER-Medicare: Brief Description of the SEER-Medicare Database (2019).

[17] Warren JL, Harlan LC, Fahey A, Virnig BA, Freeman JL, Klabunde CN, Cooper GS, Knopf KB (2002). Utility of the SEER-Medicare data to identify chemotherapy use. Med Care.

[18] Lakier JB (1992). Smoking and cardiovascular disease. Am J Med.

[19] Klepin HD, Pitcher BN, Ballman KV, Kornblith AB, Hurria A, Winer EP, Hudis C, Cohen HJ, Muss HB, Kimmick GG (2014). Comorbidity, chemotherapy toxicity, and outcomes among older women receiving adjuvant chemotherapy for breast cancer on a clinical trial: CALGB 49907 and CALGB 361004 (alliance). J Oncol Pract.

[20] Khouri MG, Douglas PS, Mackey JR, Martin M, Scott JM, Scherrer-Crosbie M, Jones LW (2012). Cancer therapy-induced cardiac toxicity in early breast cancer: addressing the unresolved issues. Circulation.

[21] Caparica R, Bruzzone M, Poggio F, Ceppi M, de Azambuja E, Lambertini M (2019). Anthracycline and taxane-based chemotherapy versus docetaxel and cyclophosphamide in the adjuvant treatment of HER2-negative breast cancer patients: a systematic review and meta-analysis of randomized controlled trials. Breast Cancer Res Treat.

[22] Almuwaqqat Z, Meisel JL, Barac A, Parashar S (2019). Breast cancer and heart failure. Heart Fail Clin.

[23] Doyle JJ, Neugut AI, Jacobson JS, Grann VR, Hershman DL (2005). Chemotherapy and cardiotoxicity in older breast cancer patients: a population-based study. J Clin Oncol.

[24] Swain SM, Whaley FS, Ewer MS (2003). Congestive heart failure in patients treated with doxorubicin: a retrospective analysis of three trials. Cancer.

[25] Luque M, Arranz F, Cueva JF, de Juan A, Garcia-Teijido P, Calvo L, Pelaez I, Garcia-Palomo A, Garcia-Mata J, Antolin S (2014). Breast cancer management in the elderly. Clin Transl Oncol.

[26] Shachar SS, Jolly TA, Jones E, Muss HB (2018). Management of triple-negative breast cancer in older patients: how is it different?. Oncology.

[27] Freyer G, Campone M, Peron J, Facchini T, Terret C, Berdah JF, Jacquin JP, Coeffic D, Hilaire Pde S, Falandry C (2011). Adjuvant docetaxel/cyclophosphamide in breast cancer patients over the age of 70: results of an observational study. Crit Rev Oncol Hematol.

[28] Bash LD, Weitzman D, Blaustein RO, Sharon O, Shalev V, Chodick G (2017). Comprehensive healthcare resource use among newly diagnosed congestive heart failure. Isr J Health Policy Res.

[29] McMurray JJ, Adamopoulos S, Anker SD, Auricchio A, Böhm M, Dickstein K, Falk V, Filippatos G, Fonseca C, Gomez-Sanchez MA (2012). ESC guidelines for the diagnosis and treatment of acute and chronic heart failure 2012: The Task Force for the Diagnosis and Treatment of Acute and Chronic Heart Failure 2012 of the European Society of Cardiology. Developed in collaboration with the Heart Failure Association (HFA) of the ESC. Eur J Heart Fail.

