# Supplementary material for: Evaluating anthracycline + taxane versus taxane-based chemotherapy in older women with node-negative triple-negative breast cancer: a SEER-Medicare study
Source: Breast Cancer Res Treat. 2021 Oct 27;191(2):389–99. doi: 10.1007/s10549-021-06424-z (PMC8763743; doi:10.1007/s10549-021-06424-z)
Supplement: Supplementary file 1 — Supplementary file1 (DOCX 16 KB) [file 10549_2021_6424_MOESM1_ESM.docx]

Supplemental Figure 1: Codes used for cardiac conditions

| Condition | ICD-9 | ICD-10 | HCPCS |
| --- | --- | --- | --- |
| Atrial Fibrillation | 42731 | I480 I481 I482 I4891 |  |
| Ischemic Heart Disease | 41000 41001 41002 41010 41011 41012 41020 41021 41022 41030 41031 41032 41040 41041 41042 41050 41051 41052 41060 41061 41062 41070 41071 41072 41080 41081 41082 41090 41091 41092 4110 4111 41181 41189 412 4130 4131 4139 41400 41401 41402 41403 41404 41405 41406 41407 41412 4142 4143 4144 4148 4149 | I200 I201 I208 I209 I2101 I2102 I2109 I2111 I2119 I2121 I2129 I213 I214 I21A1 I21A9 I220 I221 I222 I228 I229 I230 I231 I232 I233 I234 I235 I236 I237 I238 I240 I241 I248 I249 I2510 I25110 I25111 I25118 I25119 I252 I253 I2541 I2542 I255 I256 I25700 I25701 I25708 I25709 I25710 I25711 I25718 I25719 I25720 I25721 I25728 I25729 I25730 I25731 I25738 I25739 I25750 I25751 I25758 I25759 I25760 I25761 I25768 I25769 I25790 I25791 I25798 I25799 I25810 I25811 I25812 I2582 I2583 I2584 I2589 I259 |  |
| Heart Failure | 39891 40201 40211 40291 40401 40403 40411 40413 40491 40493 4280 4281 42820 42821 42822 42823 42830 42831 42832 42833 42840 42841 42842 42843 4289 | I0981 I110 I130 I132 I501 I5020 I5021 I5022 I5023 I5030 I5031 I5032 I5033 I5040 I5041 I5042 I5043 I50810 I50811 I50812 I50813 I50814 I5082 I5083 I5084 I5089 I509 |  |
| Diabetes* | 24900 24901 24910 24911 24920 24921 24930 24931 24940 24941 24950 24951 24960 24961 24970 24971 24980 24981 24990 24991 25000 25001 25002 25003 25010 25011 25012 25013 25020 25021 25022 25023 25030 25031 25032 25033 25040 25041 25042 25043 25050 25051 25052 25053 25060 25061 25062 25063 25070 25071 25072 25073 25080 25081 25082 25083 25090 25091 25092 25093 3572 36201 36202 36203 36204 36205 36206 36641 | E0800 E0801 E0810 E0811 E0821 E0822 E0829 E08311 E08319 E08321 E083211 E083212 E083213 E083219 E08329 E083291 E083292 E083293 E083299 E08331 E083311 E083312 E083313 E083319 E08339 E083391 E083392 E083393 E083399 E08341 E083411 E083412 E083413 E083419 E08349 E083491 E083492 E083493 E083499 E08351 E083511 E083512 E083513 E083519 E083521 E083522 E083523 E083529 E083531 E083532 E083533 E083539 E083541 E083542 E083543 E083549 E083551 E083552 E083553 E083559 E08359 E083591 E083592 E083593 E083599 E0836 E0837X1 E0837X2 E0837X3 E0837X9 E0839 E0840 E0841 E0842 E0843 E0844 E0849 E0851 E0852 E0859 E08610 E08618 E08620 E08621 E08622 E08628 E08630 E08638 E08641 E08649 E0865 E0869 E088 E089 E0900 E0901 E0910 E0911 E0921 E0922 E0929 E09311 E09319 E09321 E093211 E093212 E093213 E093219 E09329 E093291 E093292 E093293 E093299 E09331 E093311 E093312 E093313 E093319 E09339 E093391 E093392 E093393 E093399 E09341 E093411 E093412 E093413 E093419 E09349 E093491 E093492 E093493 E093499 E09351 E093511 E093512 E093513 E093519 E093521 E093522 E093523 E093529 E093531 E093532 E093533 E093539 E093541 E093542 E093543 E093549 E093551 E093552 E093553 E093559 E09359 E093591 E093592 E093593 E093599 E0936 E0937X1 E0937X2 E0937X3 E0937X9 E0939 E0940 E0941 E0942 E0943 E0944 E0949 E0951 E0952 E0959 E09610 E09618 E09620 E09621 E09622 E09628 E09630 E09638 E09641 E09649 E0965 E0969 E098 E099 E1010 E1011 E1021 E1022 E1029 E10311 E10319 E10321 E103211 E103212 E103213 E103219 E10329 E103291 E103292 E103293 E103299 E10331 E103311 E103312 E103313 E103319 E10339 E103391 E103392 E103393 E103399 E10341 E103411 E103412 E103413 E103419 E10349 E103491 E103492 E103493 E103499 E10351 E103511 E103512 E103513 E103519 E10359 E1036 E1037X1 E1037X2 E1037X3 E1037X9 E1039 E1040 E1041 E1042 E1043 E1044 E1049 E1051 E1052 E1059 E10610 E10618 E10620 E10621 E10622 E10628 E10630 E10638 E10641 E10649 E1065 E1069 E108 E109 E1100 E1101 E1110 E1111 E1121 E1122 E1129 E11311 E11319 E11321 E113211 E113212 E113213 E113219 E11329 E113291 E113292 E113293 E113299 E11331 E113311 E113312 E113313 E113319 E11339 E113391 E113392 E113393 E113399 E11341 E113411 E113412 E113413 E113419 E11349 E113491 E113492 E113493 E113499 E11351 E113511 E113512 E113513 E113519 E113521 E113522 E113523 E113529 E113531 E113532 E113533 E113539 E113541 E113542 E113543 E113549 E113551 E113552 E113553 E113559 E11359 E113591 E113592 E113593 E113599 E1136 E1137X1 E1137X2 E1137X3 E1137X9 E1139 E1140 E1141 E1142 E1143 E1144 E1149 E1151 E1152 E1159 E11610 E11618 E11620 E11621 E11622 E11628 E11630 E11638 E11641 E11649 E1165 E1169 E118 E119 E1300 E1301 E1310 E1311 E1321 E1322 E1329 E13311 E13319 E13321 E133211 E133212 E133213 E133219 E13329 E133291 E133292 E133293 E133299 E13331 E133311 E133312 E133313 E133319 E13339 E133391 E133392 E133393 E133399 E13341 E133411 E133412 E133413 E133419 E13349 E133491 E133492 E133493 E133499 E13351 E133511 E133512 E133513 E133519 E133521 E133522 E133523 E133529 E133531 E133532 E133533 E133539 E133541 E133542 E133543 E133549 E133551 E133552 E133553 E133559 E13359 E1336 E1339 E1340 E1341 E1342 E1343 E1344 E1349 E1351 E1352 E1359 E13610 E13618 E13620 E13621 E13622 E13628 E13630 E13638 E13641 E13649 E1365 E1369 E138 E139 |  |
| Hyperlipidemia* | 2720 2721 2722 2723 2724 | E780 E7800 E7801 E781 E782 E783 E784 E7841 E7849 E785 |  |
| Hypertension* | 36211 4010 4011 4019 40200 40201 40210 40211 40290 40291 40300 40301 40310 40311 40390 40391 40400 40401 40402 40403 40410 40411 40412 40413 40490 40491 40492 40493 40501 40509 40511 40519 40591 40599 4372 | H35031 H35032 H35033 H35039 I10 I110 I119 I120 I129 I130 I1310 I1311 I132 I150 I151 I152 I158 I159 I674 N262 |  |
| Tobacco Use* | 3051 64900 64901 64902 64903 64904 98984 | F17200 F17201 F17203 F17208 F17209 F17210 F17211 F17213 F17218 F17219 F17220 F17221 F17223 F17228 F17229 F17290 F17291 F17293 F17298 F17299 O99330 O99331 O99332 O99333 O99334 O99335 T65211A T65212A T65213A T65214A T65221A T65222A T65223A T65224A T65291A T65292A T65293A T65294A Z720 | 99406 99407 |
| Peripheral Vascular Disease* | 4400 4401 4402 44020 44021 44022 44023 44029 4404 4438 44381 44382 44389 4439 | E0851 E0852 E0951 E0952 E1051 E1052 E1151 E1152 E1351 E1352 I700 I701 I70201 I70202 I70203 I70208 I70209 I70211 I70212 I70213 I70218 I70219 I70221 I70222 I70223 I70228 I70229 I70231 I70232 I70233 I70234 I70235 I70238 I70239 I70241 I70242 I70243 I70244 I70245 I70248 I70249 I7025 I70291 I70292 I70293 I70298 I70299 I7092 I7381 I7389 I739 I791 I798 |  |

* Codes from Chronic Conditions Data Warehouse (https://www2.ccwdata.org/web/guest/condition-categories)
